# Detection of the *ADGRG6* hotspot mutations in urine for bladder cancer early screening by ARMS‐qPCR

**DOI:** 10.1002/cam4.5879

**Published:** 2023-04-20

**Authors:** Dan Tan, Wenqi Jiang, Rixin Hu, Zhuoran Li, Tong Ou

**Affiliations:** ^1^ Medical Laboratory of Shenzhen Luohu Hospital Group Shenzhen 518000 Guangdong China; ^2^ Shenzhen Following Precision Medical Research Institute of Luohu Hospital Group Shenzhen 518000 Guangdong China; ^3^ The Affiliated Shenzhen Luohu Hospital of Shantou University Medical College Shantou University Shantou 515063 China

**Keywords:** ADGRG6, ARMS‐qPCR, bladder cancer, genetic mutation, liquid biopsy

## Abstract

**Background:**

In bladder cancer, recurrent *ADGRG6* enhancer hotspot mutations (chr. 6: 142,706,206 G>A, chr. 6:142,706,209 C>T) were reported at a high mutation rate of approximately 50%. Thus, *ADGRG6* enhancer mutation status might be a candidate for diagnostic biomarker.

**Methods:**

To improve test efficacy, an amplification refractory mutation system combined with quantitative real‐time PCR (ARMS‐qPCR) assay was developed to detect the *ADGRG6* mutations in a patient as a clinical diagnostic test. To validate the performance of the ARMS‐qPCR assay, artificial plasmids, cell DNA reference standard were used as templates, respectively. To test the clinical diagnostic ability, we detected the cell free DNA (cfDNA) and sediment DNA (sDNA) of 30 bladder cancer patients' urine by ARMS‐qPCR comparing with Sanger sequencing, followed by the droplet digital PCR to confirm the results. We also tested the urine of 100 healthy individuals and 90 patients whose diagnoses urinary tract infections or urinary stones but not bladder cancer.

**Results:**

Sensitivity of 100% and specificity of 96.7% were achieved when the mutation rate of the artificial plasmid was 1%, and sensitivity of 96.7% and specificity of 100% were achieved when the mutation frequency of the reference standard was 0.5%. Sanger sequencing and ARMS‐qPCR both detected 30 cases of bladder cancer with 93.3% agreement. For the remaining unmatched sites, ARMS‐qPCR results were consistent with droplet digital PCR. Among 100 healthy individuals, three of them carried hotspot mutations by way of ARMS‐qPCR. Of 90 patients with urinary tract infections or urinary stones, no mutations were found by ARMS‐qPCR. Based on clinical detection, the ARMS‐qPCR assay's sensitivity is 83.3%, specificity is 98.4%.

**Conclusion:**

We here present a novel urine test for *ADGRG6* hotspot mutations with high accuracy and sensitivity, which may potentially serve as a rapid and non‐invasive tool for bladder cancer early screening and follow‐up relapse monitoring.

## Graphical abstract

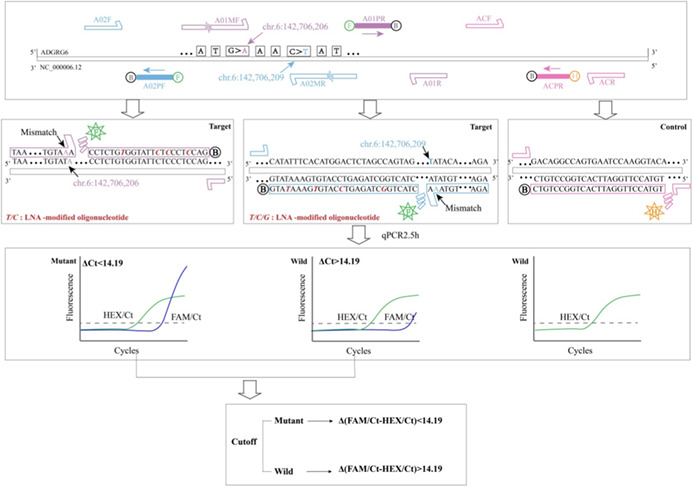

## 1 INTRODUCTION

Bladder cancer is one of the most common malignancies worldwide, with an estimated 573,000 new cases and 213,000 deaths from the disease per year worldwide, and its occurrence and development is highly correlated with genetic mutations.^1^, ^2^, ^3^ Men are around four times more likely than women to develop bladder cancer.^4^ The vast majority of bladder cancers (> 90%) are urothelial bladder carcinomas (UBCs).^5^ At the time of initial diagnosis, two forms of bladder cancer with distinct clinical and pathologic characteristics could be distinguished: two‐thirds of newly diagnosed bladder cancer cases are non‐muscle invasive, characterized by a high recurrence rate and a tendency to evolve to muscle‐invasive, and muscle‐invasive bladder cancers occurring in one‐third of patients frequently lead to distant metastases.^6^, ^7^ Survival rates between early and advanced bladder cancer are greatly different.^8^ Therefore, the early detection of bladder cancer is critical to delay progression and improving management.^9^

At present, the gold screening modalities for the initial diagnosis and follow‐up of bladder cancer is on the basis of cystoscopy associated with urinary cytology.^10^ However, they remain unsatisfactory due to high invasiveness (cystoscopy) or low accuracy (cytology). Moreover, since patients typically experience pain, discomfort, and hematuria during a cystoscopy, they are less likely to comply with the expensive scheduled control visits.^11^, ^12^, ^13^ Liquid biopsies display tremendous promise in bladder cancer and even are expected to be involved in clinical management in the future using circulating cancer cells, metabolomics, cell‐free DNA, and RNA.^14^, ^15^ Several urine biomarkers for UBC have been developed and can already be tested using commercial kits based on urinary DNA, but it is still required to further study the more sensitive and specific markers and assays.^16^, ^17^

The ideal UBC biomarker for diagnosing would produce accurate results and require little to no intrusive approach to get.^18^, ^19^ Since 2010, the improvements in patient management and outcomes are anticipated as a result of the development of various liquid biopsy analyses, which have raised the possibility that any spatial and temporal heterogeneity in tumor biology could be better tracked by serial blood analyses than by analyses of tissue samples from a primary tumor.^20^ In addition, some research has shown that DNA extracted from cells exfoliated in urine can provide information when bladder cancer is present, potentially including tumor cells that have been released from the bladder tissue.^21^, ^22^ In UBC, the telomerase reverse transcriptase (*TERT*) promoter mutations represent the most recurrent genetic mutations, which have been identified as a significant influence in carcinogenesis development for 60%–80% of bladder cancer patients.^23^, ^24^, ^25^ To date, some assays to detect urinary DNA *TERT* promoter mutations for bladder cancer early screening were established, most of them involve the use of next‐generation sequencing (NGS) platform, but the capability of rapidly detecting *TERT* promoter mutations in the clinic is restricted by its GC‐rich region.^26^, ^27^, ^28^

Therefore, a simple, efficient, and highly sensitive urinary DNA mutation test is urgently needed for bladder cancer. Growing evidence has led to an event that the adhesion G protein‐coupled receptor G6 (*ADGRG6*) enhancer mutations are high‐frequency among bladder cancer patients after *TERT*.^29^, ^30^ And most *ADGRG6* enhancer mutations occur at chr. 6: 142,706,206 G>A (Hotspot mutation 1, HM1) and chr. 6:142,706,209 C>T (Hotspot mutation 2, HM2). Hence, rapid urine‐based *ADGRG6* enhancer hotspot mutations detection holds great promise as a noninvasive approach for cancer early detection.^29^

In this study, the amplification refractory mutation system combined with quantitative real‐time PCR (ARMS‐qPCR) method was developed to rapidly and sensitively detect the specific mutations of *ADGRG6* enhancer providing a flexible and easy way to screen bladder cancer.

## 2 MATERIALS AND METHODS

### 2.1 DNA templates and clinical samples

Two mutant plasmids carried HM1 (G>A) or HM2 (C>T) were constructed by biological company, respectively (Sangon Biotech Co. Ltd.), which were confirmed by Sanger sequencing. The clones that carried HM1 (G>A) or HM2 (C>T), and *ADGRG6* wild‐type genomic DNA were used as the ARMS‐qPCR assay standards determine the sensitivity and specificity. The constructed mutant plasmids were serially diluted to 30 copy/μL in TE buffer (10 mM Tris–HCl; 1 mM ethylenediaminetetraacetic acid; pH 8.0), then 30 copy of mutant plasmid mixed with 10 ng of wild‐type cell line genome as a template for 1% mutation rate analysis (3000 haploid human genomes close to 10 ng genomic DNA approximately).

We tested 30 urine samples from patients who newly diagnosed with UBC, 90 urine samples with urinary tract infection or urinary stones but not a tumor, 100 urine samples from normal subjects in the Shenzhen Luohu People's Hospital. Urinary cell free DNA (cfDNA) and sediment DNA (sDNA) was extracted by commercial kits, and all DNA concentration and absorbance was determined by Nanodrop OneC (Thermo Fisher Scientific, Inc.). The study was approved by the Research Ethics Committee of Shenzhen Luohu People's Hospital. All the selected patients gave informed consent for this study.

### 2.2 Primers and probes design

**FIGURE 1:**
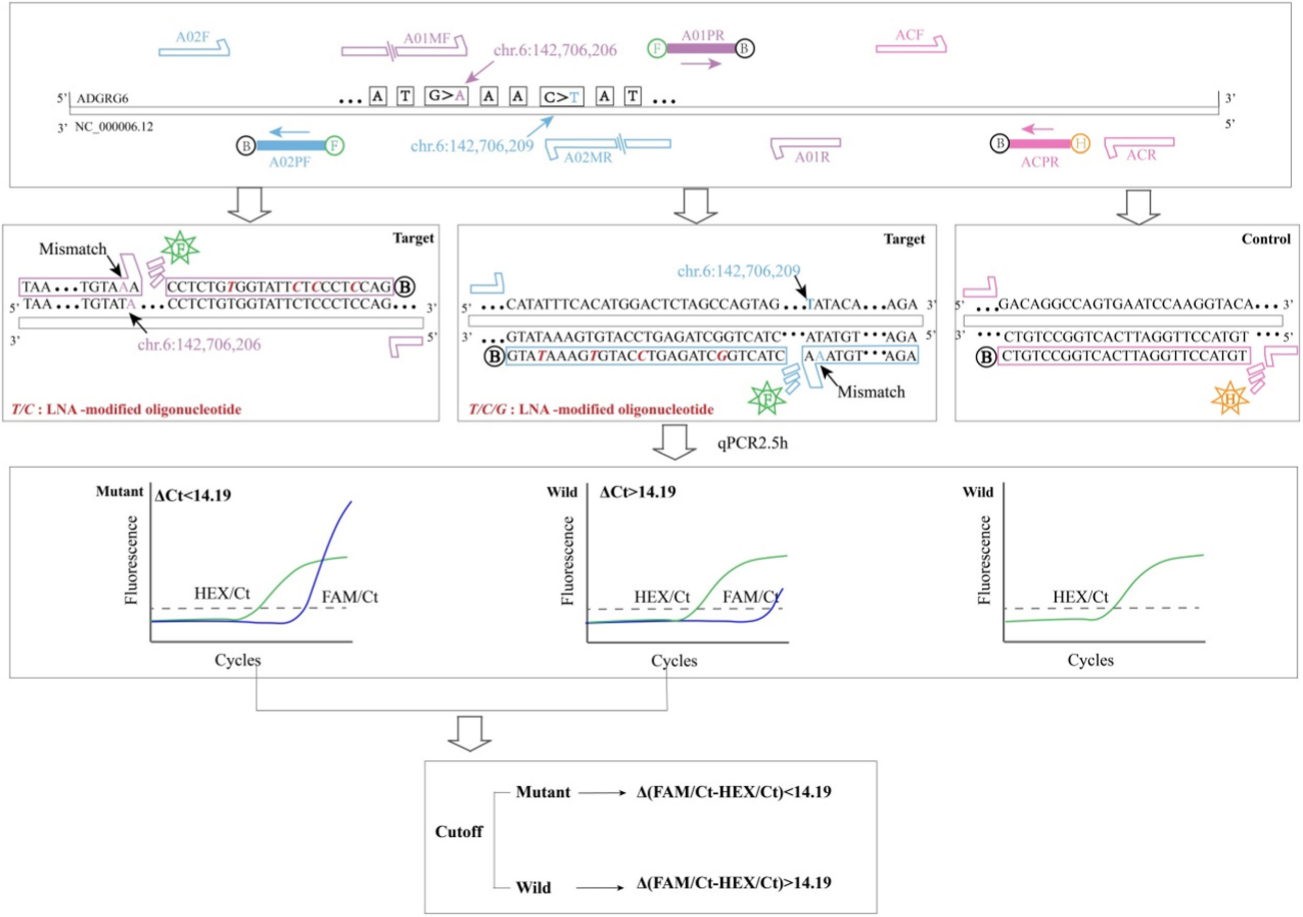
Scheme of the principle and process of the ARMS‐qPCR assay for detecting *ADGRG6* hotspot mutations in this study. Along with an explanation of how the results were interpreted, the diagram shows the relative positions of the primer‐probe sets within the *ADGRG6*, as well as primer mismatches and probe LNA‐modified oligonucleotides.

Based on two hotspot mutations in *ADGRG6* enhancer, ARMS‐qPCR primers and probes for screening UBC were designed using Primer 5.0 software. Briefly, as shown in Figure 1. Three sets of primers and probes targeting *ADGRG6* mutations and wild‐type conserved sequence fragments were shown in Table 1, and the primers and probes were synthesized at Ruibo Biotech Co. Ltd (Guangzhou, China).

**TABLE 1:** The ARMS‐qPCR primer‐probe sets used in this study.

Genotype	Name	Sequence
HM1(G>A)	A01MF	5’‐TAATTCTAACTAAGTATTTCACTCTTTGTAAA‐3′
	A01R	5’‐AGCACCGTATAAATGTTCTTGGA‐3′
	A01PF^a^	5’‐FAM‐CCTCTG */iXNA_T/* GGTATT */iXNA_C/* T*/iXNA_C/*CCT*/iXNA_C/*CAG‐BHQ‐3’
HM2(C>T)	A02MR	5’‐AGAATACCACAGAGGCTCTTTGTAAA‐3’
	A02F	5’‐CAAGGAGATTTATGATGGAGCAA‐3’
	A02PR^a^	5’‐FAM‐CTACTG*/iXNA_G/*CTAGAGT*/iXNA_C/* CATG*/iXNA_T/*GAAA*/iXNA_T/*ATG‐BHQ‐3’
Internal reference	ACF	5’‐CTGCTTATTCCATCCAGTTCA‐3’
	ACR	5’‐AGGTCCTCCAGTGTCTTC‐3’
	ACPR	5’‐HEX‐TGTACCTTGGATTCACTGGCCTGTC‐BHQ‐1‐3’

^a^ “*iXNA*” means LNA‐modified oligonucleotide.

### 2.3 Real‐time PCR

The ARMS‐qPCR performed in a 50 μL reaction mixture containing: 10 × Taq Buffer, 40 mM MgCl_2_, 2.5 mM dNTPs, 10 pM primer F(A01MF or A02F), 10 pM primer R(A01R or A02MR), 10 pM mutagenic probe(A01PF or A02PR), 10 pM internal reference primer F(ACF), 10 pM internal reference primer R(ACR), 10 pM internal reference probe(ACPR), Taq DNA polymerase(Vazyme Biotech Co., Ltd.), 10 ng of the DNA template. PCR was performed using a SLAN‐96P real‐time PCR system (Shanghai Hongshi Medical Technology Co., Ltd) under the following conditions: 95°C for 5 min, 8 cycles of 95°C for 30 s, 65°C for 30 s, 72°C for 45 s, followed by: 45 cycles of 95°C for 30 s, 60°C for 30 s, and 72°C for 40 s. The detection and measurement of a fluorescent reporter signal serves as a gauge for DNA amplification. So the determination of mutations was based on the difference between the *C*_*T*_ values targeting the unique mutations of the *ADGRG6* enhancer and the *C*_*T*_ values targeting the wild‐type conserved sequence fragment, i.e., the *ΔC*_*T*_ value.

### 2.4 HM2(C>T) reference standard

A cell DNA reference standard containing HM2(C>T) was constructed. The construction process was as follows: (1) UBC cell lines T24, SW780, 5637, and MGH were sequenced by Sanger sequencing; (2) depending on the Sanger sequencing outcome, cell lines T24, and SW780 were chosen matching wild conserved sequence and HM2 (C>T) type respectively; (3) the DNA from T24 and SW780 was extracted, and diluted to a concentration of 90 ng/μL in TE buffer preparing for subsequent quantifying using the Droplet Digital PCR (DD‐PCR) method (HM2 mutation frequency in T24 cell lines is 0, SW780 is 38.39%, Table S1); (4) on the basis of copy number quantified, the reference standard mutation frequencies of 0.1%, 0.5%, 1%, and 5% were manufactured by mixing T24 and SW780 genomic DNA diluting solution proportionally.

### 2.5 Droplet digital PCR

To quantify the copy number and mutation frequency of the two hotspot mutation sites, we designed DD‐PCR primers and probes listed in Table 2. A 20μL digital PCR reaction system was constructed and carried out on the QX200 drop digital PCR systems (Bio‐Rad Laboratories, Inc.). Each reaction mixture consisted of ddPCR Supermix for Probes, 10 pM primer, 10 pM probe, and DNA template. The PCR carried on the following conditions: pre‐denaturation 95°C, 10 min; denaturation 94°C, 30 s; annealing 60°C, 60 s, extension 98°C, 10 min, 40 cycles; and storage at 4°C. Fluorescence signals were collected for each reaction unit after amplification and at the end of the reaction procedure, the QX200 Droplet Reader was used for micro drop analysis detection and absolute quantification of DNA template based on direct counts and Poisson distribution formulae at appropriate thresholds. Based on the fluorescence signal of the probe in this method, droplets with a fluorescent signal are collectively referred to as positive droplets. Thus those with FAM fluorescence are mutant positive droplets and those with VIC fluorescence are wild‐type positive droplets.

**TABLE 2:** The droplet digital PCR primer‐probe sets used in this study.

Genotype	Name	Sequence
HM1(G>A)	D01F	5’‐TCACATGGACTCTAGCCAGTAGAT‐3’
	D01R	5’‐CAATCCTGGAGGGAGAATACC‐3’
	D01PG	5’‐VIC‐ACTCTTTGTATGAACATACA‐MGB‐3’
	D01PA	5’‐FAM‐CACTCTTTGTATAAACATACA‐MGB‐3’
HM2(C>T)	D02F	5’‐TCACATGGACTCTAGCCAGTAGAT‐3’
	D02R	5’‐CAATCCTGGAGGGAGAATACC‐3’
	D02PG	5’‐VIC‐TATGAACATACAAAGAGC‐MGB‐3’
	D02PA	5’‐FAM‐TATGAATATACAAAGAGCC‐MGB‐3’

## 3 RESULTS

### 3.1 Development of the *ADGRG6* mutation detection system

The developed method combines a quantitative real‐time PCR test with amplification refractory mutation system to identify the *ADGRG6* hotspot mutations for UBC early screening utilizing two closed‐tube reactions. The working principle of this system is illustrated in Figure 1, and the primer‐probe sets are presented in Table 1. To genotype the mutations of HM1and HM2, primers were designed with a base at the 3′ end matching the nucleic acid base of the mutant sequence so that it would amplify the mutant fragment efficiently but not amplify other variants. At the same time, we optimize sensitivity by increasing the annealing temperature to reduce non‐specific binding during amplification utilizing locking nucleic acids (LNA)‐modified oligonucleotide‐based probes for real‐time PCR. The presence of *ADGRG6* enhancer mutations (HM1, HM2) would result in a lower *ΔC*_*T*_ value compared with that of wild‐type sequence fragment.

### 3.2 Analytical studies

**FIGURE 2:**
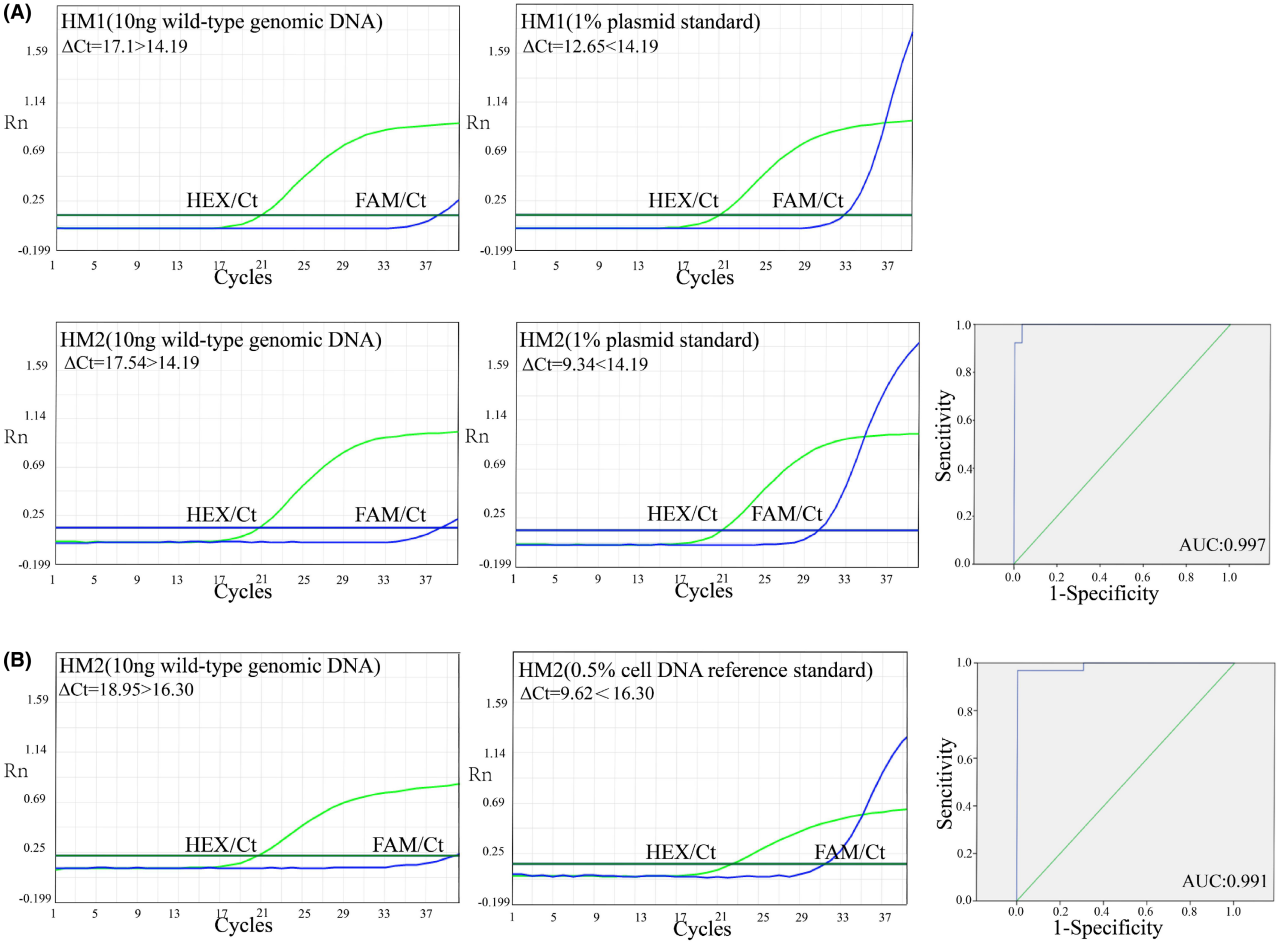
Sensitivity and specificity of the ARMS‐qPCR assays in plasmid standard with a mutation frequency of 1% (A) and HM2 (C>T) reference standard with a mutation frequency of 0.5% (B).

We performed 48 reactions of T24 cell line wild‐type DNA, 96 reactions of plasmid standards mixture with a mutation frequency of 1% (48 reactions each for both HM1 and HM2) and 48 reactions of HM2 (C>T) reference standard with a mutation frequency of 0.5%, respectively. Then, the relevant *ΔC*_*T*_ values were imported into SPSS software for ROC curve plotting analysis. It follows that the ARMS‐qPCR assay exhibits 100% sensitivity and 96.7% specificity for 1% plasmid standards. And the reference standard for the HM2 (C>T) test demonstrated that a level of 0.5% mutation ratio could be detected, indicating a sensitivity of 96.7% and a specificity of 100% (Figure 2). These results clearly revealed that the ARMS‐qPCR methods could effectively differentiate samples containing HM1(G>A) and HM2(C>T).

To define the optimum cutoff *ΔC*_*T*_ value, wild‐type genomic DNA, 1% mutation frequency of HM2(C>T) reference standard and a mixture of plasmid standards harboring either HM1(G>A) or HM2(C>T) were analyzed. With respect to the ROC curve, we select cutoff *ΔC*_*T*_ value corresponding the maximum Youden index representing optimal diagnostic utility.^31^ Finally, we choose the cutoff *ΔC*_*T*_ values to be 14.19 for HM1 and HM2 detecting (mutation frequency of 1%). If the *ΔC*_*T*_ value was less than 14.19, the sample was considered to contain the corresponding HM1 or HM2. Otherwise, if the *ΔC*_*T*_ value was larger than 14.19, the sample was considered not to contain the corresponding mutations. We also measured the cutoff *ΔC*_*T*_ value and the corresponding sensitivity and specificity of cell DNA reference standards with different mutation frequencies (Table S2).

**FIGURE 3:**
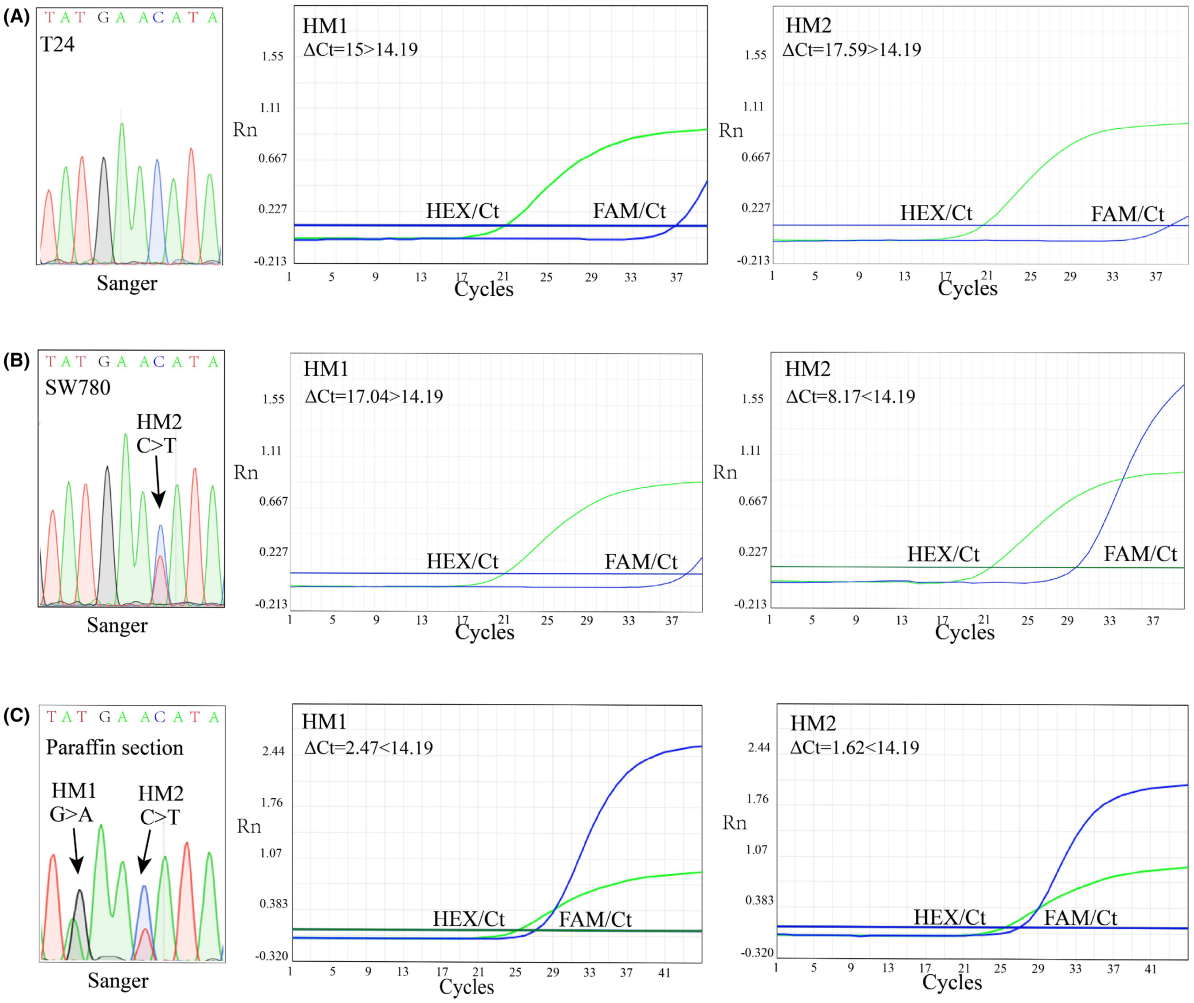
Results of ARMS‐qPCR assay and Sanger sequencing at HM1 and HM2 in T24 (A) and SW780 (B) cell lines, and one bladder cancer paraffin section example (C).

To confirm the accuracy of the system, the optimized ARMS‐qPCR assays were used for identifying the genomic DNA from UBC cell lines and 10 UBC patients' paraffin sections. The observed results revealed 100% concordance rates between ARMS‐qPCR and Sanger sequencing (Figure 3, Table S3).

To assess the reproducibility, two technicians tested three templates involving artificial plasmid templates, HM2(C>T) reference standard, and urine cfDNA on non‐consecutive seven working days by ARMS‐qPCR. Then we calculate the standard deviation of *ΔC*_*T*_ values for the three type templates were 0.37, 0.18, and 0.36, respectively (Table S4). This demonstrates the excellent reproducibility of the ARMS‐qPCR assay.

### 3.3 Performance of the ARMS‐qPCR‐based assay for clinical samples

**FIGURE 4:**
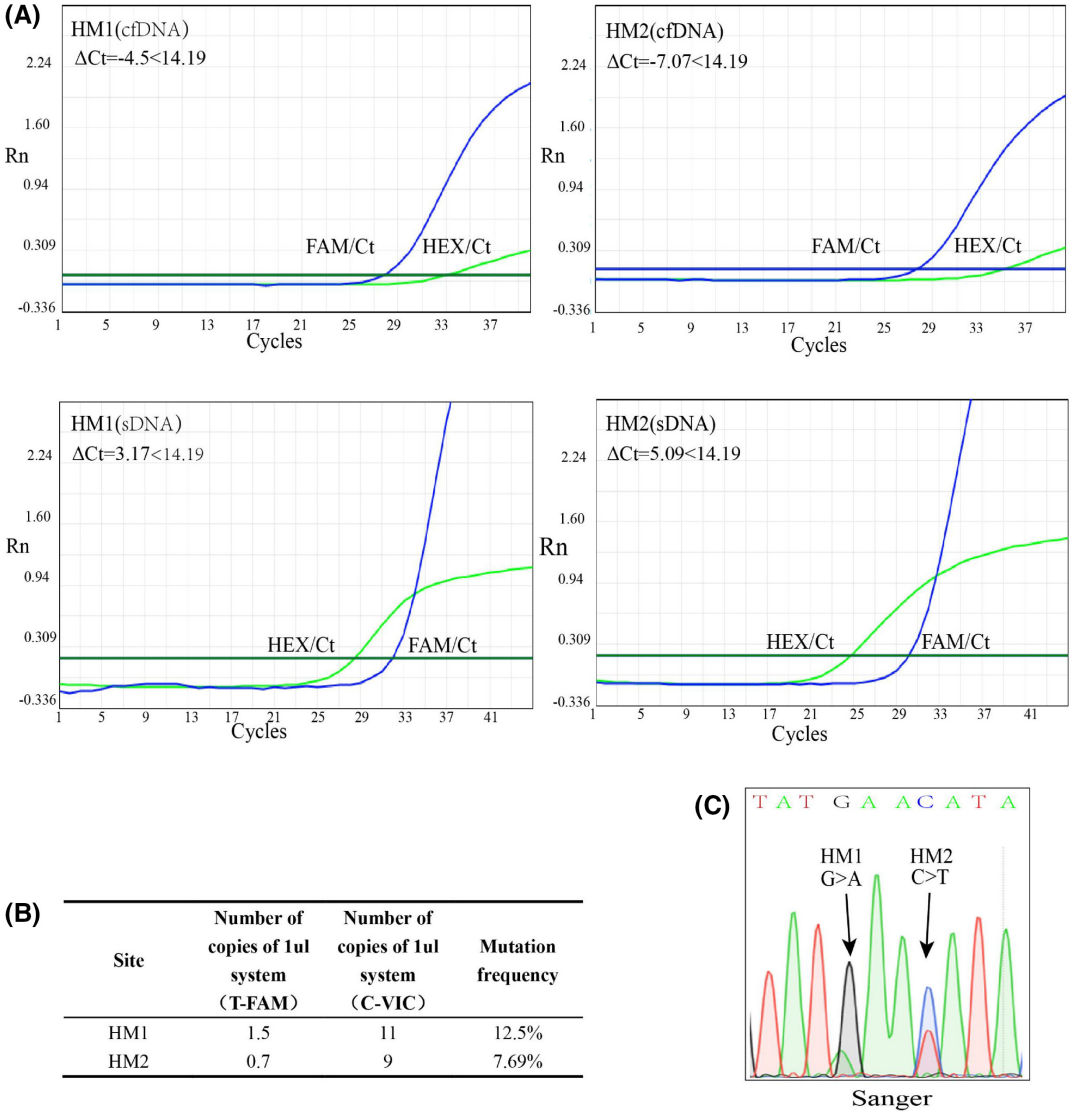
ARMS‐qPCR (A), droplet digital PCR (B) and Sanger sequencing (C) results from one bladder cancer urine sample at HM1 and HM2.

Thirty UBC patients were gathered and evaluated using urine samples to assess the ARMS‐qPCR assay's clinical performance and its potential for screening. According to the results, 25 patients (83%) had one or two hotspot mutations, including HM1 (G>A), which was identified in 19 patients, and HM2(C>T), which was identified in 15 patients. Sanger sequencing was also used to evaluate these UBC samples, and the coincidence rate between the two methods was 93.3% (56/60). DD‐PCR further detected the four samples with inconsistent results, and DD‐PCR results showed 100% agreement with ARMS‐qPCR. The above mutations from 25 patients can be detected in both urine cfDNA and sDNA (Table 3, Figure 4).

**TABLE 3:** Clinical information on bladder cancer patients and their urine DNA Sanger sequencing, ARMS‐PCR, and partial DD‐PCR test results.

NO.	Gender	Age	HM1					HM2			
			Sanger	ARMS		DD‐PCR		Sanger	ARMS		DD‐PCR
			(cfDNA)	(cfDNA)	(sDNA)	(cfDNA)		(cfDNA)	(cfDNA)	(sDNA)	(cfDNA)
U1	Female	56	Wild	G>A	G>A	0.55%		C>T	C>T	C>T	–
U2	Male	37	G>A	G>A	G>A	–		C>T	C>T	C>T	–
U3	Male	89	G>A	G>A	G>A	–		Wild	Wild	Wild	–
U4	Male	79	G>A	G>A	G>A	–		C>T	C>T	C>T	–
U5	Male	34	Wild	Wild	Wild	–		C>T	C>T	C>T	–
U6	Male	64	G>A	G>A	G>A	–		Wild	Wild	Wild	–
U7	Male	87	G>A	G>A	G>A	–		Wild	Wild	Wild	–
U8	Male	69	Wild	Wild	Wild	–		C>T	C>T	C>T	–
U9	Male	61	Wild	Wild	Wild	–		Wild	C>T	C>T	2.22%
U10	Male	78	Wild	Wild	Wild	–		Wild	Wild	Wild	–
U11	Female	80	G>A	G>A	G>A	–		C>T	C>T	C>T	–
U12	Male	67	G>A	G>A	G>A	–		C>T	C>T	C>T	–
U13	Male	76	G>A	G>A	G>A	–		C>T	C>T	C>T	–
U14	Male	60	G>A	G>A	G>A	–		Wild	Wild	Wild	–
U15	Female	82	Wild	G>A	G>A	3.33%		C>T	C>T	C>T	–
U16	Female	82	G>A	G>A	G>A	–		C>T	C>T	C>T	–
U17	Female	80	G>A	G>A	G>A	–		Wild	Wild	Wild	–
U18	Male	30	Wild	Wild	Wild	–		C>T	C>T	C>T	–
U19	Male	56	Wild	Wild	Wild	–		Wild	C>T	C>T	9.09%
U20	Male	79	G>A	G>A	G>A	–		C>T	C>T	C>T	–
U21	Male	76	G>A	G>A	G>A	–		Wild	Wild	Wild	–
U22	Female	63	G>A	G>A	G>A	–		Wild	Wild	Wild	–
U23	Male	71	G>A	G>A	G>A	–		Wild	Wild	Wild	–
U24	Male	58	G>A	G>A	G>A	–		Wild	Wild	Wild	–
U25	Male	63	Wild	Wild	Wild	–		Wild	Wild	Wild	–
U26	Female	54	Wild	Wild	Wild	–		C>T	C>T	C>T	–
U27	Male	44	Wild	Wild	Wild	–		Wild	Wild	Wild	–
U28	Male	65	Wild	Wild	Wild	–		Wild	Wild	Wild	–
U29	Male	58	G>A	G>A	G>A	–		Wild	Wild	Wild	–
U30	Male	52	Wild	Wild	Wild	–		Wild	Wild	Wild	–

Of all 100 healthy individuals, the two HM1 (G>A) and one HM2 (C>T) samples were detected through ARMS‐qPCR in urine sDNA (Table S5). The three cases were further detected by DD‐PCR and the results showed 100% concordance rates with ARMS‐qPCR (Table S6). However, only one case mutation was detectable from urine cfDNA.

Additionally, an anti‐interference test for clinical was performed on 90 specimens, which correlated with urinary tract infection or urinary stones but not a tumor (Table S5). All of the samples collected were found not to contain the HM1and HM2 mutations by ARMS‐qPCR.

To sum up, the ARMS‐qPCR assay's sensitivity is 83.3%, specificity is 98.4%, positive predictive value is 89.2%, and negative predictive value is 97.4%.

## 4 DISCUSSIONS

The diagnosis and screening of UBC currently rely on cystoscopy and urinary cytology, but cystoscopy is a costly and invasive procedure, urinary exfoliation cytology is also restricted by its sensitivity to low‐grade tumors.^32^ Bladder cancer screening methods with outstanding diagnostic efficiency and low cost are desirable in the field of clinical diagnosis.^33^, ^34^ It has proved that urine appears better at early detecting bladder cancer by liquid biopsy.^35^, ^36^ *ADGRG6* plays an important role in bladder cancer incidence and development. In the meanwhile, several findings also demonstrated its connection with regulating colorectal cancer cell proliferation and potential tumor progression in triple‐negative breast cancer.^37^, ^38^ About bladder cancer, it has a mutation rate of roughly 50%, making it the second‐most prevalent mutational hotspot in bladder cancer after the TERT promoter (70%). So the *ADGRG6* hotspot mutations may be a good clinical biomarker candidate to diagnosis in bladder cancer.^39^ For detecting, *ADGRG6* enhancer mutations were usually using Sanger sequencing or NGS, but these methods are time‐consuming or sensitivity‐limited, which may result in an underestimation of the prevalence of *ADGRG6* enhancer mutations when tumor samples contain a low percentage of mutations. To overcome these potential problems, ARMS‐qPCR uses specific probes to amplify the corresponding mutant sequences to detect mutations in specimens with as low as 1% of the mutant frequency, so that it is suitable for efficient clinical diagnosis and screening of bladder cancer.^40^, ^41^ In this study, a novel ARMS‐qPCR assay was successfully developed to detect the *ADGRG6* two hotspot mutations in two closed‐tube reactions, we used plasmids standard and reference standard demonstrating that the ARMS‐qPCR‐based method is much more sensitive (0.5% vs. 10%–15%) than conventional Sanger sequencing. At the same time, the DD‐PCR test method was designed to further prove the performance of the ARMS‐qPCR assay for two hotspot mutations. Furthermore, the cost and turnaround time of this assay was evaluated. The cost of the ARMS‐qPCR assay for each sample is 25 CNY and 96 samples can be examined within 3 h in a single run.

Through the new method, we have successfully detected corresponding *ADGRG6* enhancer fragments in urine samples from bladder cancer patients, healthy individuals and urinary tract infection or urinary stones patients, the result revealed that the ARMS‐qPCR assay's specificity is 98.4% and sensitivity is 83.3%, which would be a good test for genomic mutations screening and contribute to bladder cancer diagnosis. Therefore, the high mutation rate of *ADGRG6* enhancer hotspot mutations in bladder cancer patients in this study was attributed to the high sensitivity of the ARMS‐qPCR assay and the fact that urine allows for the better tumor information collection. According to clinical data, three additional mutant cases that were found in the screening of healthy individuals were all older than 80 years of age, this is in line with the proven evidence that the mutations is age‐related signature.^29^During the research process, both urinary cfDNA and sDNA can be used for this method, but sDNA is superior at low mutation frequency samples. It should be noted that the qPCR‐based methods can only detect known mutations, and only HM2 cell DNA reference standard was constructed and used in this study because no UBC cell line containing stable HM1 was identified in our lab.

In summary, we have developed an ARMS‐qPCR assay for detecting *ADGRG6* hotspot mutations in cfDNA and sDNA from urine samples. On the basis of results, applications of this test could be considered as a first screening step in future clinical trials for patients with high risk factors for bladder cancer, and this technology will facilitate the molecular diagnosis of UBC and likely improve early detection.

## AUTHOR CONTRIBUTIONS

**Dan Tan:** Data curation (lead); formal analysis (lead); investigation (lead); methodology (equal); project administration (supporting); resources (supporting); software (lead); validation (lead); visualization (lead); writing – original draft (lead); writing – review and editing (supporting). **Wenqi Jiang:** Data curation (supporting); investigation (supporting); methodology (supporting); resources (supporting); writing – original draft (supporting). **Rixin Hu:** Investigation (supporting); methodology (supporting); writing – original draft (supporting). **Zhuoran Li:** Resources (supporting); writing – original draft (supporting). **Tong Ou:** Conceptualization (lead); funding acquisition (lead); investigation (supporting); methodology (equal); project administration (lead); resources (lead); supervision (lead); writing – original draft (supporting); writing – review and editing (lead).

## FUNDING INFORMATION

This study was supported by the Guangdong Basic and Applied Basic Research Foundation (2022A1515220005), the Shenzhen Science and Technology Program (JCYJ20210324121613037), and the Shenzhen Key Medical Discipline Construction Fund (SZXK054).

## CONFLICT OF INTEREST STATEMENT

None.

## ETHICS STATEMENT

The study was approved by the Research Ethics Committee of Shenzhen Luohu People's Hospital and informed consent was obtained from the participants.

## Supporting information


**Table S1.** Genomic DNA copy number and mutation frequency at the HM2 site in T24 and SW780 cell lines.
**Table S2.** Different cutoff *ΔC*_*t*_ corresponding to HM2 reference standard with different mutation frequencies.
**Table S3.** Sanger sequencing and ARMS‐qPCR results of UBC cell lines and paraffin sections.
**Table S4.** Two technicians (A and B) tested artificial plasmid (template‐1), HM2 reference standard (template‐2), and clinical sample (template‐3) over a non‐consecutive period of seven working days.
**Table S5.** Clinical information relating to the patients with urinary tract infection or urinary stones and healthy population in this study
**Table S6.** Further quantification by droplet digital PCR (DD‐PCR) of some normal individuals’ urine samples detected mutations by ARMS‐qPCR.

## Data Availability

All data supporting this study are contained within the article or Supplementary tables, or available from the corresponding authors on reasonable request.
