# Supplementary material for: Detection of the ADGRG6 hotspot mutations in urine for bladder cancer early screening by ARMS‐qPCR
Source: Cancer Med. 2023 Apr 20;12(10):11503–12. doi: 10.1002/cam4.5879 (PMC10242345; doi:10.1002/cam4.5879)
Supplement: Supplementary file 1 — Table S1. Genomic DNA copy number and mutation frequency at the HM2 site in T24 and SW780 cell lines. Table S2. Different cutoff ΔC t corresponding to HM2 reference standard with different mutation frequencies. Table S3. Sanger sequencing and ARMS‐qPCR results of UBC cell lines and paraffin sections. Table S4. Two technicians (A and B) tested artificial plasmid (template‐1), HM2 reference standard (template‐2), and clinical sample (template‐3) over a non‐consecutive period of seven working days. Table S5. Clinical information relating to the patients with urinary tract infection or urinary stones and healthy population in this study Table S6. Further quantification by droplet digital PCR (DD‐PCR) of some normal individuals’ urine samples detected mutations by ARMS‐qPCR. [file CAM4-12-11503-s001.docx]

**Supplementary table 1** Genomic DNA copy number and mutation frequency at the HM2 site inT24 and SW780 cell lines.

| **Cell line** | **Number of copies of 1ng genomic DNA** | | **Mutation frequency** |
| --- | --- | --- | --- |
|  | **T-FAM** | **C-VIC** |  |
| T24 | 0 | 308 | 0 |
| SW780 | 138 | 223 | 38.39% |

**Supplementary table 2** Different cutoff *ΔC_t_* corresponding to HM2 reference standard with different mutation frequencies.

| **Mutation frequency** | **Cutoff (*ΔC_t_*)** | **Sensitivity (%)** | **Specificity (%)** |
| --- | --- | --- | --- |
| 0.1% | 18.78 | 87 | 75 |
| 0.5% | 16.30 | 96.7 | 100 |
| 1% | 14.2 | 100 | 100 |
| 5% | 8.55 | 100 | 100 |

**Supplementary table 3** Sanger sequencing and ARMS-qPCR results of UBCcell lines and paraffin sections.

| **Template** | **HM1** | |  | **HM2** | |
| --- | --- | --- | --- | --- | --- |
|  | **ARMS** | **Sanger** |  | **ARMS** | **Sanger** |
| T24 | Wild | Wild |  | Wild | Wild |
| SW780 | Wild | Wild |  | C＞T | C＞T |
| Example-1 | G＞A | G＞A |  | C＞T | C＞T |
| Example-2 | G＞A | G＞A |  | C＞T | C＞T |
| Example-3 | G＞A | G＞A |  | C＞T | C＞T |
| Example-4 | G＞A | G＞A |  | Wild | Wild |
| Example-5 | G＞A | G＞A |  | C＞T | C＞T |
| Example-6 | Wild | Wild |  | C＞T | C＞T |
| Example-7 | Wild | Wild |  | C＞T | C＞T |
| Example-8 | Wild | Wild |  | Wild | Wild |
| Example-9 | G＞A | G＞A |  | C＞T | C＞T |
| Example-10 | G＞A | G＞A |  | Wild | Wild |

**Supplementary table 4** Two technicians (A and B) tested artificial plasmid (template-1), HM2 reference standard (template-2), and clinical sample (template-3) over a non-consecutive period of 7 working days.

| **Day** | **Template-1** | |  | **Template-2** | |  | **Template-3** | |
| --- | --- | --- | --- | --- | --- | --- | --- | --- |
|  | **A(*ΔC_T_*)** | **B(*ΔC_T_*)** |  | **A(*ΔC_T_*)** | **B(*ΔC_T_*)** |  | **A(*ΔC_T_*)** | **B(*ΔC_T_*)** |
| 1 | 11.78 | 11.9 |  | 5.52 | 5.76 |  | 7.75 | 7.9 |
| 3 | 11.48 | 11.12 |  | 5.57 | 5.61 |  | 8.76 | 8.52 |
| 5 | 10.75 | 10.93 |  | 5.58 | 5.65 |  | 8.7 | 8.5 |
| 7 | 11.34 | 11.92 |  | 5.72 | 5.57 |  | 8.12 | 7.98 |
| 9 | 11.19 | 11.44 |  | 5.74 | 5.64 |  | 8.15 | 8.21 |
| 11 | 11.64 | 11.77 |  | 5.49 | 4.99 |  | 8.79 | 8.93 |
| 13 | 11.08 | 11.36 |  | 5.59 | 5.60 |  | 8.42 | 8.47 |

**Supplementary table 5** Clinical information relating to the patients with urinary tract infection or urinary stones and healthy population in this study

| **Group** |  | **Number** |
| --- | --- | --- |
| **Anti-interference group** | **Age** |  |
|  | 20-40 | 35 |
|  | 40-60 | 19 |
|  | 60-80 | 18 |
|  | 80-100 | 18 |
|  | **Gender** |  |
|  | Male | 28 |
|  | Female | 62 |
|  | **Diagnosis** |  |
|  | Urinary tract infection | 52 |
|  | Urinary stones | 38 |
|  | **Total** | **90** |
| **Healthy population** | **Age** |  |
|  | 20-40 | 7 |
|  | 40-60 | 18 |
|  | 60-80 | 58 |
|  | 80-100 | 17 |
|  | **Gender** |  |
|  | Male | 50 |
|  | Female | 50 |
|  | **Total** | **100** |

**Supplementary table 6** Further quantification by droplet digital PCR(DD-PCR) of some normal individuals’ urine samples detected mutations by ARMS-qPCR.

| **NO.** | **Gender** | **Age** | **ARMS-qPCR** | |  | **DD-PCR** | |
| --- | --- | --- | --- | --- | --- | --- | --- |
|  |  |  |  |  |  | **(Mutation frequency)** | |
|  |  |  | **HM1** | **HM2** |  | **HM1** | **HM2** |
| NU1 | Female | 90 | G＞A | Wild |  | 28.76% | 0 |
| NU2 | Female | 81 | G＞A | Wild |  | 22.73% | 0 |
| NU3 | Male | 82 | Wild | C＞T |  | 0 | 11.11% |
